# Mesenchymal Stromal Cell Secretome for the Treatment of Immune-Mediated Inflammatory Diseases: Latest Trends in Isolation, Content Optimization and Delivery Avenues

**DOI:** 10.3390/pharmaceutics13111802

**Published:** 2021-10-27

**Authors:** Elena Munoz-Perez, Ainhoa Gonzalez-Pujana, Manoli Igartua, Edorta Santos-Vizcaino, Rosa Maria Hernandez

**Affiliations:** 1 NanoBioCel Research Group, Laboratory of Pharmaceutics, School of Pharmacy, University of the Basque Country (UPV/EHU), Paseo de la Universidad 7, 01006 Vitoria-Gasteiz, Spain; elena.munozp@ehu.eus (E.M.-P.); ainhoa.gonzalez@ehu.eus (A.G.-P.); manoli.igartua@ehu.eus (M.I.); 2 Biomedical Research Networking Centre in Bioengineering, Biomaterials and Nanomedicine (CIBER-BBN), Institute of Health Carlos III, 28029 Madrid, Spain; 3 Bioaraba, NanoBioCel Research Group, 01006 Vitoria-Gasteiz, Spain

**Keywords:** MSCs, secretome, hydrogel, extracellular vesicles, exosomes, immunomodulation, IMIDs, IBD, GvHD, OA

## Abstract

Considering the high prevalence and the complex pharmacological management of immune-mediated inflammatory diseases (IMIDs), the search for new therapeutic approaches for their treatment is vital. Although the immunomodulatory and anti-inflammatory effects of mesenchymal stromal cells (MSCs) have been extensively studied as a potential therapy in this field, direct MSC implantation presents some limitations that could slow down the clinical translation. Since the beneficial effects of MSCs have been mainly attributed to their ability to secrete a plethora of bioactive factors, their secretome has been proposed as a new and promising pathway for the treatment of IMIDs. Formed from soluble factors and extracellular vesicles (EVs), the MSC-derived secretome has been proven to elicit immunomodulatory effects that control the inflammatory processes that occur in IMIDs. This article aims to review the available knowledge on the MSC secretome, evaluating the advances in this field in terms of its composition, production and application, as well as analyzing the pending challenges in the field. Moreover, the latest research involving secretome administration in IMIDs is discussed to provide an updated state-of-the-art for this field. Finally, novel secretome delivery alternatives are reviewed, paying special attention to hydrogel encapsulation as one of the most convenient and promising strategies.

## 1. Introduction

Immune-mediated inflammatory diseases (IMIDs) are pathological conditions characterized by defective immune responses that lead to chronic tissue damage [1]. This group of chronic and highly disabling conditions that share inflammatory pathways includes inflammatory bowel disease (IBD), osteoarthritis (OA) or graft-versus-host-disease (GvHD), among others [1,2,3]. The prevalence of some of these pathologies is reaching epidemic proportions, causing a huge economic impact on health-care systems worldwide [4].

The pharmacological management of IMIDs is complex, since current treatments are mainly symptomatic and generally based on corticosteroids, biological therapies or immunosuppressants in the most severe cases [5]. The frequent resistance to these drugs together with their important side effects importantly limit their clinical use [6]. Thus, intense research is taking place to develop new therapeutic strategies for the treatment of IMIDs. Among them, therapies based on mesenchymal stromal cells (MSCs) represent a novel and promising alternative [7]. Over the last decades, intense debate has taken place regarding the most adequate terminology and phenotypic characterization for MSCs, which is discussed in Box 1 [8,9,10,11,12,13,14,15,16,17].

Initially, the interest in MSCs was based on their ability to differentiate into stromal tissue cells and their engraftment capacity. Indeed, their regenerative effects have been explored in many studies from the very beginnings of this research field. However, it soon became apparent that, once implanted in the body, cells did not survive long enough to engraft and differentiate into tissue cells, whereas, they were able to produce and release bioactive factors to their microenvironment exerting important effects [18,19]. Indeed, the set of bioactive factors produced by MSCs—also known as the MSC-derived secretome—has been demonstrated to modulate the exacerbated or erratic immune responses observed in IMIDs by regulating cells from both innate and adaptive immunity [20]. Thus, MSC therapies have evolved into a new picture frame. Their grafting capacity has taken a back seat, and, instead, attention has now shifted to the secretory properties. It is not the cells for tissue construction that are required, but the beneficial effects of the released factors that are pursued [21]. Since reaching that inflection point, the secretory properties of MSCs have been thoroughly studied [19,22].

It is important to mention that MSCs present a high immunoplasticity and hence, the content of their secretome varies depending on the cell microenvironment [23]. By means of toll-like receptors (TLRs) present in their membrane, MSCs detect the cytokines present in their surroundings. It has been widely reported that TLR4 activation with factors such as LPS activates the MSC1 phenotype, in which they mostly elaborate proinflammatory mediators. In contrast, factors such as interferon γ (IFN-γ), tumor necrosis factor α (TNF-α) or poly-I:C result in TLR3 activation and MSC polarization towards an MSC2 phenotype leading to the secretion of mediators that have been proven to regulate and control the function of immune effector cells and lead to tissue repair and homeostasis [4,24,25,26]. Therefore, this MSC2 phenotype represents a promising avenue for the treatment of IMIDs, in which there is an exacerbated/erratic immune response.

**Figure 1:**
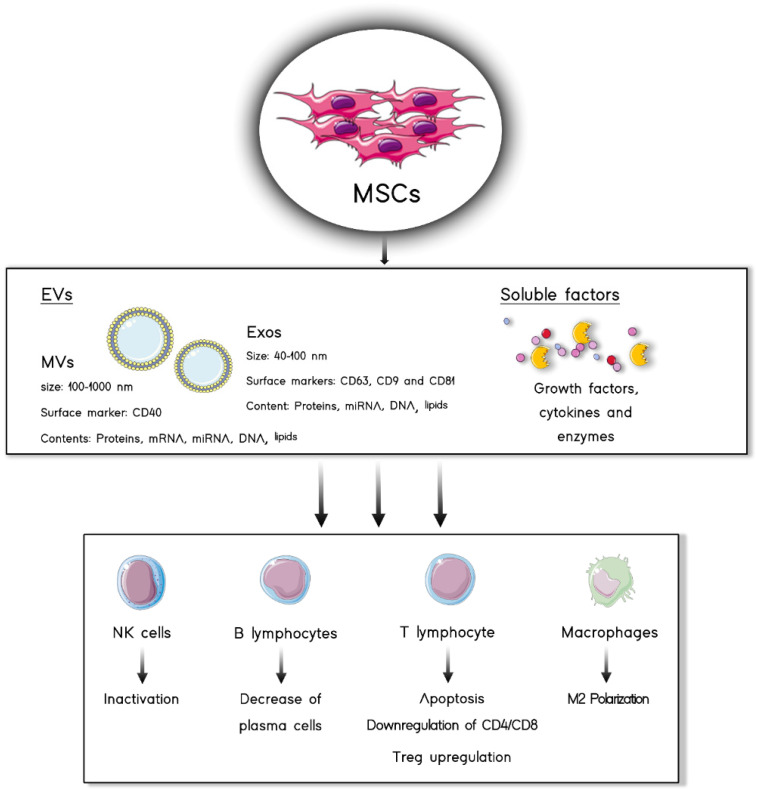
MSC secretome is composed of two different fractions: EVs and soluble factors. Both of them are capable of eliciting immunomodulatory effects on immune cells such as lymphocytes, macrophages and NK cells.

However, despite the intense research in the field and the important advances, there are still some limitations in the therapeutic use of MSCs (Figure 1). One of these issues is the lack of protocol standardization. Discrepancies among published results are frequently attributed to variances in cell source, cell isolation and culture techniques or administration routes [22]. Another important concern is the poor retention and survival of the implanted cells. It has been reported that less than 1% of MSCs survive more than one week after systemic administration [27]. This therapeutic window may be insufficient in the majority of the applications [27,28]. Furthermore, to obtain an adequate secretion profile that serves as a treatment of IMIDs, MSCs have to present an immunomodulatory MSC2 phenotype. However, in many IMIDs, the chronic inflammation levels reached are not sufficient to polarize the cells towards such a phenotype and their production of immunomodulatory factors is very limited, compromising the efficacy of the therapy [3].

Regarding safety, although intravenous administration of human MSCs is generally accepted as safe, only showing postinfusion febrile and site reactions, the likelihood of more severe side effects such as thrombosis or adverse inflammatory effects is still present [18,22,27,29,30]. Another obstacle for the clinical translation of cell-based therapies is that their legal regulation is complex and not well defined in some of the cases.

Box 1 **MSC designation and identification.**

In a series of studies performed between the 1960s and 1980s, Friedenstein and co-workers isolated adherent cells from mouse bone marrow (BM) which formed fibroblast-like colonies and were capable of generating bone tissue when transplanted [8,9,10,11]. Posterior studies demonstrated that these cells were also able to generate cartilage and adipose tissue, and that they were present in embryonic and adult tissues such as umbilical cord, adipose tissue, skin or dental pulp [11,13]. In the late 1990s the term “mesenchymal stem cells” was first used by Arnold Caplan. MSC definition has been controversial over the years because of a lack of unified criteria for their terminology and characteristics. Thus, terms like “mesenchymal stem cells”, “marrow stromal cells”, “multipotent stromal cell” or “mesenchymal stromal cells” have been used arbitrarily. To tackle this problem, in 2005, by means of a position statement, the International Society for Cellular Therapy (ISCT) established the term “mesenchymal stromal cells” as the most suitable for the MSCs acronym [15]. A year later, the ISCT proposed a list of phenotypic characteristics that identify MSCs [15]. In this context, the ISCT stated that MSCs are plastic-adherent cells that grow forming fibroblast-like colonies and present the ability to differentiate into three different cell lineages: osteoblasts, chondroblasts and adipocytes. Moreover, according to the ISCT, MSCs are characterized by the presence of surface markers including CD73, CD90 and CD105; and the absence of CD45, CD34, CD14 or CD11b, CD79a or CD19 and class II histocompatibility complex antigen (HLA-DR) [14]. Nevertheless, these criteria have been widely criticized for not being accurate enough and the correct MSC defining criteria and designation is still polemic. In general terms, the ISCT guidelines have been taken as an updateable basic knowledge about MSCs, useful for defining them but not enough to describe them completely [8].

Because of these issues—and taking into account that the secretory effects of MSCs are considered the main factor responsible for their therapeutic properties [27,28]—the attention has now shifted to the MSC-derived secretome, which will be deeply reviewed in Section 2. The use of the secretome derived from MSCs instead of the cells themselves can overcome some of the important limitations above mentioned. One of the key advantages of secretome-based therapies is that they resolve multiple safety concerns. The secretome is not a living proliferative cell population and this avoids the potential adverse effects that could occur with the implantation of cells. Furthermore, related to safety, the danger of tumorigenicity related to cryopreservants used for cell storage has vanished [31]. On the other hand, while MSC implantation involves the unknown production of bioactive factors, the MSC-derived secretome can be evaluated for content prior to its administration [29]. This contributes to protocol standardization since it enables measurement of the dosage and consequently, the obtained results are more scalable and reproducible [27]. Moreover, this overcomes the issue of the MSC-phenotype, since cells can be in vitro preconditioned to present an immunomodulatory-MSC2 phenotype and collect the resulting enriched, conditioned media. Considering all this, it is possible to say that cell-free therapies based on the MSC-derived secretome open a new path to safer therapies that are scalable, standardizable and easier to regulate [3].

## 2. MSC-Derived Immunomodulatory Secretome

**Figure 2:**
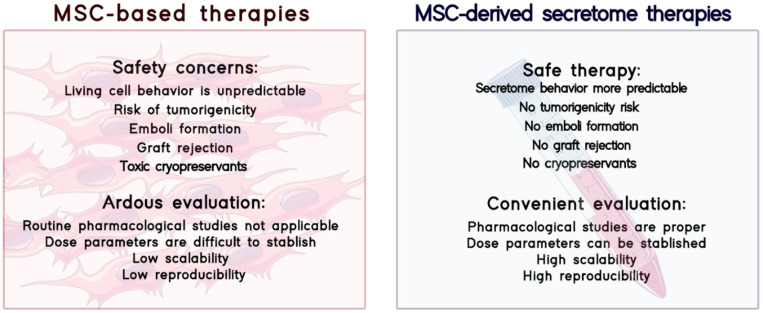
Comparison between MSC-based therapies and MSC-derived secretome therapies in terms of safety and evaluation of therapies.

MSCs can mediate immunomodulation through cell−cell interactions by means of membrane ligands, such as programmed death-ligand 1 (PD-L1) and FAS ligand (FAS-L), which bind to inhibitory receptors on immune cells [32,33]. However, as previously mentioned, the bioactive factors that MSCs secrete have been recognized as the main factors responsible for their effects [26]. The set of bioactive factors released by these cells to the extracellular space is known as MSC-derived secretome [34]. In a descriptive approach, this secretome presents two differentiated parts: a first one formed from soluble factors and a second one composed of extracellular vesicles (EVs) [35] (Figure 2). In this section, we will revise the composition of the secretome derived from the MSC2 phenotype.

### 2.1. Soluble Factors

Among the soluble factors secreted by the anti-inflammatory MSC2 phenotype, it is possible to find a plethora of molecules including growth factors, cytokines and enzymes. In the following lines, some of the most relevant factors will be discussed, highlighting the effects that make them potential candidates for the treatment of IMIDs [36].

Prostaglandin-E2 (PGE2) has been described as one of the soluble factors of the MSC secretome [3]. The release of this anti-inflammatory factor has been related to natural killer (NK) cell inhibition, macrophage polarization towards an M2 phenotype, suppression of T cell proliferation and the promotion of regulatory T cell (Treg) differentiation [37,38,39].

Another important group of factors in the soluble MSC-derived secretome are galectins, which are involved in cell–matrix adhesion, cell–cell interactions and transmembrane signaling. Furthermore, some galectins produced by MSCs have also been demonstrated to present immunomodulatory properties, as is the case of galectin 1 (Gal-1) and galectin 9 (Gal-9). The former has been reported to inhibit T cell proliferation and to regulate cytokine release, whereas the latter is involved in the inhibition of T and B cells [40,41].

Indoleamine 2, 3-dioxygenase (IDO) is a cytosolic metabolic enzyme that can also be secreted [7]. IDO catabolizes tryptophan and the depletion of this amino acid causes T cell anergy. Moreover, in this metabolic pathway, toxic kynurenine metabolites are produced, which exert a cytotoxic action on effector T cells and inhibit NK and B cells [40]. Furthermore, IDO stimulates macrophage polarization to the M2 phenotype, characterized by IL-10 production which inhibits T cell proliferation and promotes Treg differentiation [1,2,3,4,5,6,8,10,11,12,14,15,16,17,18,19,20,21,22,23,24,25,26,27,28,29,30,31,32,33,34,35,36,37,38,39,42,43,44,45].

Another interesting factor released by MSCs is interleukin 1 (IL-1) receptor antagonist (IL-1Ra), which when bound to IL-1 receptors impedes the proinflammatory effects of this cytokine. Thus, among others, IL-1Ra limits CD4^+^ T cell activation, helper T cell (Th) differentiation, B cell proliferation and macrophage IL-1β secretion [46,47,48].

TNF-α-induced gene/protein 6 (TSG-6) is another secretory factor responsible for the anti-inflammatory activity exerted by MSCs [49]. Indeed, it has been demonstrated to mediate immunosuppressive effects on multiple immune cells. Among them, TSG-6 is able to inhibit neutrophil migration [50,51], to suppress Th1 immune responses [52] and to promote Treg production [51]. Regarding macrophages, TSG-6 limits TNF-α secretion by activated macrophages [53,54] and promotes macrophage polarization towards an anti-inflammatory M2 phenotype [55,56]. As another example, leukemia inhibitory factor (LIF) produced by MSCs has also been related to the immunomodulatory effects of the secretome [57].

Among the multiple growth factors released by MSCs there are some with described immunomodulatory effects. One of the most remarkable is transforming growth factor β (TGF-β) because of its ability to inhibit Th activation, NK proliferation and cytotoxic activity and dendritic cell (DC) maturation [58,59,60]. Moreover, TGF-β also promotes the differentiation of T cells into Tregs [60]. Similarly, hepatocyte growth factor (HGF), also present in secretome, is a pleiotropic factor especially relevant for its ability to inhibit T cell proliferation and DC activity and stimulate Treg differentiation [61,62].

### 2.2. EVs

The second fraction of the MSC-derived secretome is composed of EVs. EVs are membrane-packed vesicles that act as a system of intercellular communication. They consist of a lipid bilayer that envelops bioactive molecules including lipids, proteins or nucleic acid species [35]. The lipid bilayer is not only an envelope since it is enriched with proteins like traspanins, integrins and other ligands that allow the traffic, adhesion and endocrine effects of EVs [34,35,36]. MSC-derived EVs are classified into three groups attending mainly to their biogenesis mechanism, size and surface markers.

Apoptotic bodies are the biggest ones with a size of ≥1000 nm, they are formed by cell apoptosis processes and usually express cell death markers such as Caspase 3 or Annexin V [34].

The second group are microvesicles (MVs)—also known as shedding vesicles—which are formed by the budding of the plasma membrane of the source cells. The formation process requires the reorganization of the cytoskeleton and the liberation is intracellular calcium dependent. Their diameter varies from 100 to 1000 nm and their density goes from 1.04 to 1.07 g/mL [63]. Surface markers in MVs include CD40 and their membrane is usually enriched in lipids such as cholesterol, sphingomyelin and ceramide [63]. The MV cargo is formed mainly by mRNAs and miRNAs and their interaction with target cells occurs by specific receptor−ligand interactions between MVs and target cells [36].

The third group are exosomes, which have an endosomal origin [64]. They are formed via the endocytosis−exocytosis pathway [36]. Briefly, exosome formation starts when a small amount of intracellular fluid is included in an early endosome [65]. This early endosome matures and expands into a late endosome, which can merge with other endosomal membranes to form intraluminal bodies—also known as multivesicular bodies (MVBs) [66]. The exosomes are finally formed when MVBs are released to the extracellular environment by exocytosis [67]. The release process is regulated by proteins including p53 and TSG101 and controlled by the cytoskeleton activation pathway but it is not affected by calcium signaling [29,36]. As previously mentioned, they have a size of 40–100 nm and its density goes from 1.13 to 1.19 g/mL [66]. As the exosomes present bilayered lipid membranes they can also include small amounts of phosphatidylserine and high amounts of cholesterol, sphingolipids and ceramides [36]. They normally express CD9, CD63, CD81 and CD79 markers as well as clathrins, anexines and other biomarkers [68]. When the exosomes are released to the extracellular environment, their main objective is to establish communication with other cells [36]. The interaction between exosomes and target cells can occur by three different mechanisms, such as direct fusion of the exosomes with cell membrane or endocytosis resulting in the transmission of the content to the target cell intracellular milieu. This transmission can also occur by the interaction between the lipid ligand receptors present on cells and the lipids on the exososomal membrane [36,64,69]. Their cargo includes mostly genetic material such as DNA or miRNA noncoding fragments as well as proteins [64,70,71].

Interestingly, the cargo of the last two groups—MVs and exosomes—has been proven to elicit major cellular effects. Their mRNA content has been suggested to elicit direct influence on cell differentiation and proliferation, while the miRNA cargo has been reported to modulate their phenotype. Indeed, several miRNA contained in EVs have been reported to present immunoregulatory and regenerative properties [29,72,73]. In their protein fraction, EVs can contain multiple the soluble components of the secretome aforementioned, presenting important immunomodulatory effects [29,74,75]. Considering all the above mentioned, the EV fraction of the secretome itself is a potential candidate for the treatment of IMIDs [34].

## 3. Secretome Isolation

Once centrifuged to remove cell debris, the expended medium where the MSCs are cultured, usually known as “conditioned medium” (CM) contains both fractions of the MSC-derived secretome [63]. According to the latest research, the EV fraction could mediate an important effect in the management of IMIDs and therefore, more and more attention is being paid to its isolation, since it implies the concentration of the bioactive components [35,76].

**Figure 3:**
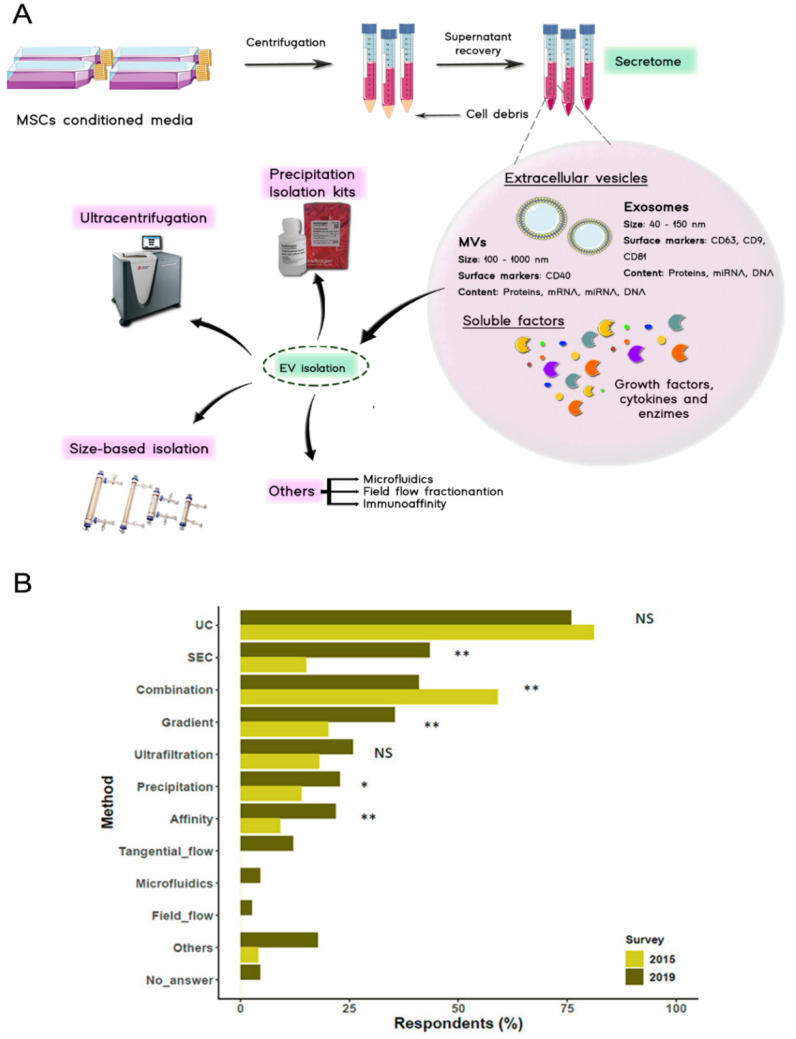
(**A**) Secretome isolation starts with a centrifugation stage where cell debris are pelleted and discarded, after that, secretome fractions can be isolated by employing different techniques such as centrifugation, size-based isolation techniques or commercial kits among others. (**B**) Results of the survey carried out by the ISCT regarding the techniques used for the isolation of EVs in 2019. Ultracentrifugation and a combination of size-based isolation techniques are the most commonly used protocols for EV isolation. (NS, non-significant, * *p* < 0.05, ** *p* < 0.01. Missing bars: not queried in the 2015 survey) (Adapted with permission from [82]).

The gold standard technique for EV isolation is ultracentrifugation (Figure 3) [35,77]. The described isolation protocols recommend a series of centrifugations of the CM to remove cell debris, followed by ultracentrifugation to pellet the EVs [35,78,79]. Although ultracentrifugation has been demonstrated to isolate EVs, it is a time-consuming technique and it is limited by the risk of disruption of EV integrity [80]. Furthermore, problems may appear during the process as it is a laborious protocol and the aggregation of the EVs could easily occur due to gravitational forces [35,81]. Moreover, during the centrifugation of the CM, the soluble factors of the secretome may also become deposited [82]. This event could open a discussion as to whether the therapeutic effects of the EVs are solely related to the effect of the MVs and exosomes [35].

Size-based isolation techniques have also been very useful. Ultrafiltration devices, tangential flow filtration (TFF) systems or size exclusion chromatography (SEC) have turned out to be interesting strategies [82,83]. Blind-elution chromatography has returned good results in the purification of the EVs in combination with a prefiltration with TFF devices [80].

Commercialized rapid isolation kits are also widely employed for EV isolation [80]. All of them are based on the isolation by precipitation methods. Some of the available kits are ExosQuick (System Biosciences), Exos-spin (Cell Guidance Systems) or Total Exosome Isolation Reagent (Thermo Fisher, Waltham, MA, USA). Unfortunately, although commercial kits are useful for separating EVs from the soluble fraction of the secretome, the achieved purity is often rather poor [79]. Less employed techniques include affinity-based capture [77]. For example, immunoaffinity methods have been used for the identification and isolation of specifically marked EVs. This method could be useful if specific EVs are required for the intended therapy [77].

Nowadays, in laboratory practice, ultracentrifugation and size exclusion techniques are often combined since ultracentrifugation enables vesicle isolation and size exclusion techniques to allow their purification. However, many efforts are being made to optimize and gain knowledge on these procedures, since it is well known that the isolation method affects the amount, type and purity of EVs as well as their effect [35]. Furthermore, isolation and purification techniques could be useful to concentrate the bioactive compounds, which may be beneficial for lowering therapeutic dose volume and therefore, to obtain more manageable treatments. Despite the advances made so far, there is still a need for isolation techniques that present a high yield, maintain the characteristics of the obtained products and are not cost prohibitive but scalable [63].

## 4. Factors That Introduce Variability in the Secretome Composition

### 4.1. Donor Source

In many studies where MSC-based therapy is explored, autologous cells are harvested from donor patients to avoid cell rejection. Nevertheless, factors such as the age or health condition of the donor have been shown to pose a great influence on the growth, development and immunomodulatory capacity of the primary MSCs [84]. Indeed, the secretome has been shown to vary its composition depending on these factors. For example, the secretome obtained from donors with multiple sclerosis had less neuroprotective effect in comparison to that of healthy patients [85]. Likewise, obesity of donors has shown to have a great influence on the composition of the cargo of the EVs derived from adipose tissue MSCs (AT-MSCs), reducing the concentration of macrophage inflammatory protein (MIP) and miRNA-126 [86]. This effect could be limited by using allogeneic sources. However, primary MSCs—both autologous or allogeneic—are less long-lived and their characteristics and behavior change throughout the cell passages. Similarly, donor age has an impact on the MSC secretory profile. Aging has been associated with MSC senescence which can lead to an important modification of their secretome, especially in terms of miRNA. Indeed, senescent cells adopt a different secretory profile known as senescent-associated secretory phenotype (SASP). As well as differences in content, aging cells have been reported to present a higher EV production as well as EV size reduction [87,88]. This means that the composition of the MSC-derived secretome can be changeable and not reproducible.

### 4.2. Cell Source

Another factor that presents a direct effect on the composition of the MSC-based secretome is the cell tissue source. The differences in the secretome originated from alternative cell sources has been evidenced thanks to proteomic studies. One of them showed that there is a significant difference in the protein profile released from bone marrow (BM-MSCs), AT-MSCs and dental pulp-derived MSCs. However, the secretion of proteins that have been related to the therapeutic potential of the secretome is constitutive in all the cellular sources analyzed so far [89]. In this way, the differences in secretome from these different tissues are mainly quantitative with a greater or lesser variability on the concentration of the secreted bioactive compounds depending on the cell source. Backing up these results, studies comparing the secretome protein expression of human MSCs derived from BM, Wharton’s jelly (WJ) and exfoliated deciduous teeth (SHED) demonstrated quantitative differences in a variety of growth factors and cytokines. In particular, IL-10 expression in the secretome was higher in WJ-MSCs and SHED-MSCs while the latter was also enriched in HGF [90]. In another comparative study, the secretome derived from WJ-MSCs, AT-MSCs and BM-MSCs was demonstrated to have different concentrations of the proteins of interest. In particular, the WJ-MSC-derived secretome was enriched in factors like RANTES, IL1-Ra TGF-β, HGF and IL-6 [91,92].

It is important to mention that variability introduced by the cell donor or origin makes the MSCs unpredictable and as a result, there is an increasing interest in their replacement by immortalized cell lines [93]. According to the latest research, immortalized MSC-derived secretome does not vary depending on tissue source or cell passage [74,94]. Furthermore, since an important quantity of cells are required for an optimal yield of secretome production, the use of immortalized MSCs with lower cost and better longevity results is appealing. All of these considerations show the way to new prospects for the use of immortalized MSCs lines in secretome-based therapies.

To date, the effect of cell source on EV composition has not been directly explored. Instead, a few studies have addressed it indirectly by comparing the global therapeutic effects obtained by EVs originated from alternative tissue sources. In a comparative study, exosomes obtained from menstrual MSCs (mMSCs) presented greater therapeutic effects on neurodegenerative pathologies like Parkinson’s disease when compared to BM, umbilical cord and chorion-derived MSCs [95]. Considering that tissue source definitely introduces variability in the whole secretome composition, it is highly likely that it also affects the EV fraction. Therefore, in EV-based therapies, exploring this avenue would shed light on which could be the source presenting EVs with a better therapeutic profile for the treatment of certain diseases.

### 4.3. MSCs Preconditioning Strategies: Towards an Msc2 Anti-Inflammatory Secretome

The possibility of manipulating the MSCs’ microenvironment to polarize them towards an MSC2 phenotype that renders a secretome loaded with anti-inflammatory factors is currently in vogue. In the following sections, we review some of the most explored strategies that could be implemented in secretome-derived therapies for the treatment of IMIDs.

#### 4.3.1. Hypoxia

The influence of controlled oxygen exposure when culturing MSCs in vitro has been largely studied [96,97,98]. Normally, in vitro cell culture is carried out under an 18–21% oxygen concentration. However, it has been reported that hypoxic culture conditions, with an oxygen tension varying between 0–10% created using a cell-culture chamber or bioreactors, could enhance the secretion of the immunomodulatory factors by MSCs. Indeed, hypoxic preconditioning has been demonstrated to increase MSC production of pleiotropic growth factors such as HGF, IGF-1 or brain-derived neurotrophic factor (BDNF) and immunomodulatory factors such as IDO or PGE2, among others [99,100,101,102,103]. In a study conducted by Yu et al., the secretome derived from hypoxia preconditioned MSCs exerted an immunomodulatory effect on microglia cells modulating their proinflammatory cytokine secretion and promoting their polarization towards an anti-inflammatory phenotype [104]. These effects were attributed to a secretome enriched in factors such as IL-10, HGF or PGE2 [105].

According to the latest research, EV concentration and cargo could also vary when hypoxic preconditioning is employed. Hypoxic conditions have been reported to not alter the average size, morphological appearance and surface markers of MSC-derived EVs [106,107]. Not so well established is whether hypoxia could promote or demote EV production by MSCs. Despite some studies reporting that EVs release could be triggered by culturing MSCs in hypoxic conditions [104], others revile this hypothesis, advocating the opposite [25,107]. This controversy might be based on the use of different hypoxic conditions, since slight changes in oxygen concentration and exposure time may have an important impact [104]. Regarding the therapeutic effect of hypoxia preconditioned EVs, it has been observed that they are capable of exerting immunomodulatory effects by enhancing macrophage polarization through M2 anti-inflammatory phenotypes [25,108]. It has been proposed that EVs could enrich their cargo when preconditioned, this being the principle role of their miRNA cargo [109,110,111]. Among others, miRNA-21 has been suggested as a key mediator on preconditioned EVs’ immunomodulatory effects [108].

The mechanisms involved in these effects have not been accurately described yet. However, it has been reported that hypoxia induces the secretion of hypoxia-inducible factor (HIF-1), which influences cell metabolism, differentiation, proliferation, migration and survival [97]. In addition, there are indications that the induction of HIF-1 is directly related to the mechanisms of membrane fusion and budding and therefore in the release process of EVs [112,113,114,115,116]. Moreover, it is believed that since in vivo damaged tissues are frequently hypoxic (1–7%), the culture of MSCs in this environment increases their viability after being implanted [3,117]. Nevertheless, it is important to note that whereas some studies have observed increased cell survival after exposure to hypoxic conditions [118], others have reported cytotoxic effects [119]. These disparate outcomes could be due, again, to differences in the severity and exposure time of the hypoxic conditions [3,120].

#### 4.3.2. Three-Dimensional (3D) Culture

Even though MSCs are normally cultured in monolayers, 3D cultures have been shown to enhance immunomodulatory factor production by MSCs [27]. There are several strategies for MSC 3D culture, one of the most used is spheroid formation [53,121]. In this scenario, less oxygen reaches the inner layer cells, providing a hypoxic microenvironment, which together with the promotion of cell−cell contacts, has been reported to enhance immunomodulatory factor secretion [53]. Recent studies report a higher secretion of granulocyte—colony stimulating factor (G-CSF) and IL-1Ra by MSCs when cultured in spheroids. In addition, the secretome obtained from 3D cultured MSCs (3D-MSC-secretome) has been proven to stimulate macrophage polarization preventing the release of proinflammatory factors such as TNF-α and stimulating their IL-10 secretion [53,122,123]. Furthermore, an increase in anti-inflammatory factors such as PGE2 has also been observed [53,124]. However, this approach could limit the viability of grafted cells since their inner core could present oxygen and nutrient diffusion limitations [121]. Efforts now focus on 3D-MSC-secretome characterization and, although not intense, research has been performed in this regard, first approaches have already been carried out. Specifically, the 3D-MSC-secretome has been characterized and compared to the secretome obtained from 2D-cultured MSCs (2D-MSC-secretome) demonstrating that the spheroid culture of MSCs could lead to an enrichment of the obtained secretome profile on anti-inflammatory factors such as IL-10. In contrast, the 2D-MSC-secretome presented higher levels of pro-inflammatory cytokines such as IL-6 or IL-2 [125]. In terms of the EV fraction of the secretome, it has been described that 3D-cultured MSCs have a higher efficiency on EV production compared to 2D-cultured MSCs. Moreover, EVs obtained from 3D-cultured MSCs (3D-MSC-EVs) have been shown to display superior functions in some studies [126,127].

Other strategies such as cell culture on scaffolds have also been employed for 3D-MSC-secretome obtainment. This method has been demonstrated to have a direct impact on secretome composition and function increasing its HGF levels [128]. However, one of the most interesting approaches for obtaining a 3D-MSC secretome is the encapsulation of MSCs in hydrogels [129,130]. Hydrogels enable the immediate cell microenvironment to be tuned in terms of mechanical properties—elasticity, stiffness—and also, to decorate it with motifs present in the natural extracellular matrix, all of which contribute to secretome enrichment [131]. This new approach has recently been combined with the biochemical stimulation of cells prolonging the immunomodulatory MSCs phenotype [132,133].

#### 4.3.3. Biochemical Stimuli

In recent years, the exposure of MSCs to biochemical stimuli has been widely explored. This process is also known as licensing. As previously mentioned, it has been described that TLR-3 activation with inflammatory cytokines such as IFN-γ, TNF-α or IL-1 polarizes MSCs towards an MSC2 anti-inflammatory phenotype. As a result, exposing MSCs to these factors has become a widely used strategy on therapies based on the MSC-derived secretome for the treatment of IMIDs [134,135].

For instance, cell exposure to IFN-γ has been proven to unfetter the production of factors such as HGF, Gal-9, IDO or PGE2, thus eliciting immunomodulatory and regenerative effects [7,27,63,136,137]. Similarly, MSC licensing with TNF-α has been related to secretome enrichment with tissue repairing and immunomodulatory growth factors [138,139]. Significantly, this enrichment is greater when proinflammatory stimuli with TNF-α is combined with hypoxic conditions during preconditioning [107,140]. Interestingly, the combination of both cytokines, IFN-γ and TNF-α, has been demonstrated to have a synergistic effect to polarize MSCs towards the MSC2 phenotype [44].

In this vein, recent studies have shown that exposure to IL-1β and TNF-α enriches the secretome in proteins such as granulocyte-macrophage colony stimulating factor (GM-CSF), monocyte chemoattractant protein-1 (MCP-1) and matrix metalloproteinases 8 and 9 [107]. Interestingly, other studies have demonstrated that IL-1β not only enriches the factors above mentioned but also HGF and IL1-Ra, which exert important immunomodulatory effects [141].

Variations in the EV-secretion profile have also been studied after MSC exposure to biochemical stimuli. It has been shown that IFN-γ is capable of inducing minor changes on EV shape and size when being used in high concentrations [142]. However, these changes do not always occur [106,107] and there is still the need to confirm if minor modifications on EV size would affect their therapeutic potential. The concentration of EVs released by licensed MSCs has also been shown to be higher. In particular, AT-MSCs were demonstrated to release a higher quantity of EVs when subjected to proinflammatory stimuli. However, although this increase in the secretion of EVs points to a greater therapeutic effect, it is vital to characterize the effect of licensing on the EV cargo. In this regard, recent research has demonstrated that the proinflammatory licensing of MSCs also enhances the immunomodulatory effects of EVs. In a study performed by Serejo et al., EVs derived from AT-MSCs licensed with IFN-γ inhibited T-cell proliferation to a higher extent than unlicensed EVs. These effects were attributed to an increase of IDO and Gal-1 within the EV cargo [143]. Similar effects have been described when licensing MSCs with TNF-α and IL-1β [143,144,145].

Although IFN-γ, TNF-α and IL-1 are the most studied factors, some others have also been demonstrated to boost the EV immunomodulatory effects. For instance, the use of thapsigargin has also been tested. The resultant WJ-MSC-derived EVs showed an increased yield and expression of TGFβ, cyclooxygenase 2 (COX2) and IDO compared to control EVs. Moreover, licensed EVs exerted direct immunomodulatory effects by suppressing T-cell proliferation and differentiation while increasing Treg proliferation and macrophage M2 polarization. Remarkably, these immunomodulatory effects of the EVs obtained by licensed MSCs were related to therapeutic outcomes observed in dextran sodium sulfate (DSS)-induced colitis mice models [146].

## 5. Application of the MSC Secretome for the Treatment of IMIDs

Considering the therapeutic potential of the MSC-derived secretome for the treatment of IMIDs, its use in animal models is being extensively studied.

### 5.1. IBD

IBD is a chronic IMID, which comprises ulcerative colitis and Crohn’s disease. Characteristic of IBD is the inflammation of the gastrointestinal tract, which causes several symptoms such as diarrhea, bloody stool and pain spikes. The pathology of IBD has been related to immune disbalances such as excessive T cell activation, reduction of Treg differentiation and overproduction of proinflammatory mediators in the gastrointestinal tract [147]. Consequently, the immunomodulatory properties of the MSC-derived secretome have become an interesting new avenue for its treatment.

Both local or systemic administration of MSC-derived EVs in dextran sodium sulphate (DSS)-induced mice models have been demonstrated to improve the clinical severity of colitis, improving survival rates and showing less gut inflammation on histopathological studies [148,149,150]. These beneficial properties of the EVs have been related to the polarization of macrophages towards an M2 anti-inflammatory phenotype, as well as to oxidative stress reduction in the affected tissues when being administered by intraperitoneal injection or locally into injured tissue through laparotomy [150,151]. Preconditioning the MSCs with IFN-γ and TNF-α has also been demonstrated to enrich the EVs in immunomodulatory proteins such as HGF, PGE2 or TGF-β eliciting superior effects when administered by intraperitoneal injection [148]. Similarly, MSC-derived CM has been proven to downregulate the expression of proinflammatory cytokines such as TNF-α or IL-1 and upregulate the anti-inflammatory factors such as IL-10 in DSS-induced colitis mice models [152].

A different strategy that has also been explored is the in vitro exposure of macrophages to the MSC-derived secretome. Once implanted in a 2,4,6-Trinitrobenzenesulfonic acid (TNBS)-induced colitis mice model, preconditioned macrophages were demonstrated to improve animal survival rate by decreasing weight loss and bloody stool, and thus alleviating colitis progression and avoiding disease recurrence [153].

### 5.2. OA

OA is a disease that causes the progressive degeneration of joints [154,155,156]. It is characterized by cartilage degradation, calcification and inflammation processes that lead to symptoms such as reduced movement capacity, joint stiffness and diffuse pain [154]. Remarkably, OA has been related to poor cartilage formation−degradation balance with low rates of chondrocyte proliferation and a notable cell apoptosis [154]. Moreover, immune disbalances such as macrophage overactivation have also been described in OA. Interestingly, the MSC-derived secretome has been studied as a new therapeutic approach for the treatment of OA showing promising results in preclinical animal models [154,157].

In a series of studies performed in OA mice models, an exosome intra-articular injection was shown to have both protective and therapeutic effects in the damaged joints [155,156]. MSC-derived exosomes have also been demonstrated to prevent cartilage and bone from degradation improving disease severity [154]. Moreover, exosomes have been proven to elicit regulatory effects in chondrocytes, increasing their proliferation and migration rates as well as enhancing their type II collagen synthesis, which improves cartilage mechanic resistance [155,158]. It is remarkable that these beneficial effects have been attributed to the immunomodulatory activity of exosomes including the upregulation of anti-inflammatory mediators such as IL-10, IL-13 or IL-4, the downregulation of proinflammatory mediators such as TNF-α or IL-1 and the polarization of macrophages towards their M2 anti-inflammatory phenotype, among others [154,159,160].

Indeed, it has been observed that MSC-derived CM, as well as isolated EVs are able to ameliorate OA symptoms by preventing joint deterioration and improving tissue remodeling in mice joint disease models [161]. Finally, it is also worth mentioning that MSC preconditioning strategies, such as the 3D culture of MSCs, increases the effectiveness of secretome-based treatments in OA, accomplishing higher synthesis rates of glycosaminoglycan (GAG) in damaged cartilage as well as recovering chondrocyte proliferation and migration [125].

### 5.3. GvHD

Hematopoietic stem cell transplantation (HSTC) is a clinical curative approach for the treatment of hematological malignancies. When HSCT is performed, GvHD can become a risky limitation. GvHD occurs when donor T cells respond to the human leucocyte antigens (HLA) of the host cells. This incompatibility between donor and host cells results in immune system disorders with varied clinical manifestations of disease on the skin, liver or gastrointestinal tract, among others [162].

The immunomodulatory properties of MSCs were first reported to be useful for treating GvHD caused by HSCT in 2004 [163]. Since such promising findings, several studies, including both preclinical and human clinical trials have extensively evaluated the use of MSCs for the treatment of GvHD. However, results are varying and controversial [164,165]. This has mainly been attributed to heterogeneity of protocols that include disparities in cell source and dosage, and could also be caused by the ability of the specific microenvironment in each specific patient to polarize the anti-inflammatory MSC2 phenotype. In this scenario, the use of cell-free therapies could represent an interesting alternative [3].

Indeed, EVs have shown promising outcomes in the treatment and prevention of GvHD [162]. Clinical manifestations of both acute and chronic GvHD have been proven to be milder when employing MSC-EVs as a treatment. Among them, skin, liver and gastrointestinal disease symptoms have been reverted and controlled [166]. Recently, Dal collo et al. tested the effects of intraperitoneally administered BM-MSC-derived EVs on an in vivo mice model of acute GvHD [139]. The results demonstrated that EVs were not only capable of improving disease severity but also of preventing its development, increasing survival of the animals [139].

Furthermore, in acute GvHD mice models, MSC-derived EVs administered intravenously have been proven to modulate cytokine production, enhancing IL-10 production and controlling TNF-α, IL-17 and IL-2 expression, which are typically related to the inflammatory stages in GvHD [167].

Isolated exosomes themselves have also been tested on GvHD models. As Lai and coworkers demonstrated in their research with GvHD mouse models, MSC-derived exosomes intravenously injected decreased proinflammatory cytokine secretion as well as being demonstrated to elicit T cell activation in host bone marrow and spleen [168,169].

Preclinical studies have shown promising results in pathologies such as IBD, OA or GvHD (Table 1).

### 5.4. Coronavirus Disease 2019 (COVID-19) and Acute Respiratory Distress Syndrome (ASDR)

COVID-19 caused by severe acute respiratory syndrome coronavirus 2 (SARS-CoV-2) is a contagious respiratory disease declared a pandemic on 11 March, 2020. Symptoms of COVID-19 include fever, cough, fatigue, dyspnea and smell sense loss. The viral infection produces a torrent release of proinflammatory cytokines leading, in many cases, to fatal outcomes and the death of patients. One of the common complications in cases of coronavirus infection is the acute respiratory failure known as acute respiratory distress syndrome (ARDS). Physiopathologically, ARDS occurs when there is continuous damage to the pulmonary epithelium. This triggers an excessive inflammatory response in the tissue, weakening it and causing edema and dysfunction. This excessive immune response has been related to the presence of proinflammatory cytokines and the activation of macrophages and neutrophils in the pulmonary alveoli [170]. Due to this altered immunological component in ARDS, the immunomodulatory effect of MSCs could be especially beneficial for the treatment of COVID-19 cases with this complication. Indeed, a clinical trial on allogenic MSCs on severe COVID-19 patients demonstrated the therapy to be beneficial for COVID-19-induced ARDS. In the study, five patients were injected with the treatment and the clinical symptoms of ARDS improved dramatically [171]. In fact, the results in this trial were so encouraging that the compassionate use of MSCs of severe COVID-19 cases has been recently approved by the FDA. However, additional research is required in larger population samples.

Regarding cell-free therapy based on the MSC-derived secretome, recent studies have revealed that it could also have therapeutic potential in COVID-19/ARDS treatment. Indeed, in preclinical studies of ARDS, MVs have been reported to be a valuable strategy. Worthy of mention, an endotoxin-induced mouse model of acute lung inflammation (ALI) showed a reduction in lung-edema when MVs were instilled intra-tracheally and similar results were shown in lipopolysaccharide ALI-induced mice. The mechanism behind these effects has not been elucidated yet, but it is believed that part of the mRNA cargo of MVs, such as the Ang-1 mRNA or KFG mRNA, is related to the therapeutic effects.

The effect of MSC-derived exosomes has already been studied in a phase I clinical trial of COVID-19 induced ARDS. In the trial, 24 severe COVID-19 patients presenting ARDS were intravenously injected 15 mL of BM-MSC-derived exosomes with a non-specified dose. The treatment was demonstrated to improve the survival of patients as well as to control neutrophil and macrophage lung infiltration and lung edema appearance, in comparison to the status prior to exosome treatment. Moreover, no adverse effects were detected during the trial and patient survival was determined to be above the 80% [172].

## 6. Secretome Delivery

The immunomodulatory effects of the MSC-derived secretome have been widely demonstrated. However, the immediate challenge in this field is still to find a safe, effective and controlled manner for its delivery [173]. In the treatment of IMIDs, as in many other pathologies, it is especially important to obtain a controlled and sustained release of bioactive factors [174]. Nude secretome administration has not been successful in many cases [175]. When administered systemically, a significant fraction is clarified and removed by macrophages, that is to say, permanence is very limited [175,176,177,178,179]. Similarly, topically administered nude secretome also showed a low bioavailability [173,175]. These facts do not only impair the effectiveness of the treatment but also increase their cost [180,181]. Secretome obtainment, especially if EV isolation is required, is arduous and large-scale production has not yet been achieved with reproducibility [182,183]. Thus, secretome delivery needs to have a higher yield and dose wastage should be avoided.

Encapsulation of the secretome into administration delivery matrices is a possible solution to these problems [184,185]. One of the most interesting strategies may be hydrogel encapsulation, since it could enable a sustained and controlled release extending the half-life of the secretome and decreasing its administration frequency [186]. As a result, lower secretome production would be needed and dosage availability would be higher, enhancing also patient comfort and compliance [173,175].

Hydrogels are three-dimensional, hydrophilic, porous and soft matrices that present high water content [187]. They are formed by the crosslinking of natural—e.g., chitosan, hyaluronic acid (HA) or alginate—or synthetic—e.g., poly-ethylene glycol (PEG) or poly-lactic-glycolic acid (PLGA)—polymers with crosslinking agents [175]. Natural polymers can be also distinguished by their source since they can be extracted from human or non-human sources such as other animals, plants or bacteria. Unlike other strategies, hydrogels are easily produced [188] and they can be formulated under mild conditions—room temperature and moisture conditions, gentle stirring, no need for cell-toxic chemicals. This is of paramount importance for the encapsulation of thermosensitive, brittle or breakable factors [189]. Furthermore, it is possible to elaborate biocompatible hydrogels by using the adequate polymers [173,190]. Moreover, the final products are manageable and easily modifiable for comfortable administration [187,191]. In this regard, the formulation of injectable hydrogels enables the in situ crosslinking, which once administered, adapt to and fill the space of the local milieu. Therefore, the encapsulation of the different fractions of the MSC-derived secretome in hydrogels is a promising alternative for their delivery [192]. In that sense, special interest has been paid to the hydrogel encapsulation of the EV-fraction of the secretome.

**Figure 4:**
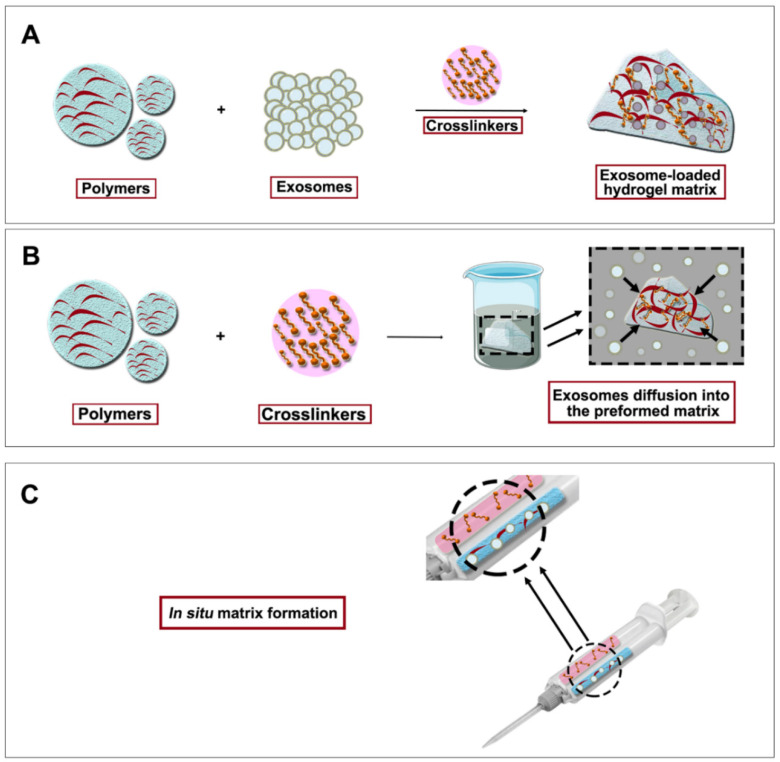
Exosome-loaded hydrogel formation strategies. (**A**) Exosomes are first mixed with polymers and then crosslinkation is performed. (**B**) Breathing method where hydrogel matrix is first built up and exosomes are allowed to diffuse into the matrix. (**C**) In situ matrix formation where hydrogel components and exosomes are simultaneously mixed with the use of a double syringe system.

Different hydrogel encapsulation strategies have been used for EV delivery, which vary in polymer composition—including both natural and synthetic polymers—and the encapsulation technique. According to the encapsulation techniques, EVs can be included in the hydrogel before, after or at the same time as the crosslinkers [173] (Figure 4). The order of encapsulation will depend mostly on the selected polymers and the porosity of the produced matrices. An example illustrating the first technique where polymers are mixed up with EVs before the crosslinker addition is the system designed by Quin et al. They developed an EV-loaded hydrogel made of thiolated hyaluronic acid, thiolated heparin and thiolated gelatin with polyethylene glycol diacrylate (PEGDA) as a crosslinker. This process of mixing EVs and polymers before adding the crosslinkers is the most employed as it is simple and suitable for the majority of the polymers employed [193].

The “breathing” encapsulation method has also been employed for EV encapsulation in hydrogels [173]. Briefly, polymers and crosslinkers are mixed and afterwards, the formed matrix is submerged into an EV solution, resulting in their entrance into the hydrogel matrix. This breathing is suitable for porous systems since EVs would have to cross their matrix for the encapsulation [194].

Following the third approach, a polypeptide hydrogel made of Pluronic F127, oxidative hyaluronic acid, and poly-ε-L-lysine was developed by Wang and co-workers [195]. In this case, polymers, crosslinkers and EVs were mixed at the same time. This mixture order allows the in situ gelation of the hydrogel and its immediate administration in vivo. Simultaneous mixing can be used when polymer gelation is quick and a rapid encapsulation of EVs is needed [195].

The release of EVs from hydrogels can occur through different mechanisms, including diffusion if the matrix porosity allows it, polymer-EV interactions and matrix degradation. Therefore, changes in factors such as hydrogel composition, the chemical nature of its components, the crosslinking method used for its gelation or even the environment where the hydrogel is administered will directly affect the EV release and thus, the performance of the system. Furthermore, the mechanical properties of hydrogels can be a determining factor in the administration of the formulations, making it more or less difficult. Commonly, a shear-thinning behavior of the hydrogels is desirable—being less viscous when administered but recovering elasticity and shape when remaining in steady state—which enables manageable administration of treatments and increased permanence rates on injured areas [196].

Apart from the hydrogels containing the EV-fraction, whole secretome hydrogel encapsulation has also been explored so far. In this vein, one of the most promising approaches is the combination of the MSC preconditioning culture strategies for secretome enrichment and the subsequent secretome hydrogel encapsulation for its delivery into injured areas [176]. An example of this strategy is the one employed by Waters et al., which employs AT-MSC-3D culture to enrich their secretome in regenerative factors. Hydrogel encapsulation of the obtained secretome was employed as a successful treatment in cardiac infarcted mouse models [197]. Interestingly, the most explored strategies for secretome encapsulation are both the breathing and the in situ crosslinking methods because of their simplicity. However, and as it happens, in EV-encapsulation, the followed strategy depends mostly on the polymers used for hydrogel formation [176,198].

However, even if secretome hydrogel encapsulation has been demonstrated to be promising, this field is in its infancy [199]. Despite hydrogels being widely employed in cell therapy, their use in secretome delivery has not yet been fully exploited [200,201,202]. Indeed, as shown in Table 2, most of the studies in which EVs or secretome are encapsulated in hydrogels are focused on regenerative medicine applications such as osteoporosis, endometrial tissue repair, hind limb ischemia, wound healing or cardiac tissue repair [155,195,203,204,205,206,207,208,209,210,211,212,213,214,215,216]. Therefore, future research is guaranteed to explore the potential of this approach for the delivery of immunomodulatory secretome for the treatment of IMIDs.

## 7. Concluding Remarks

Despite numerous studies showing that the immunomodulatory effects of MSCs are promising in inflammation management, the cell-based therapies present important limitations. Concerns regarding cell administration—including implant rejection, risk of thrombosis or adverse inflammatory reactions, as well as the difficult reproducibility and bioactive factor evaluation—have put MSC-based therapies at stake. Several studies show that the immunomodulatory effects of MSCs can be mainly attributed to their secretome, which has led to a turning point where cellular therapies could be replaced by cell-free therapies. In this sense, the MSC-derived immunomodulatory/anti-inflammatory secretome is the new promising vein of gold, overcoming the drawbacks of cell-based therapies. This is encouraging the scientific community to study its therapeutic effects and the interest in the field is increasing rapidly.

Regarding its application, using secretome as a treatment on IMIDs such as IBD, osteoarthritis or GvHD has been demonstrated to elicit therapeutic effects. However, there is still a great research pathway ahead since there are still limitations for this therapeutic potential to overcome. First, an exhaustive characterization of secretome is necessary to ensure comprehension and reproducibility of the treatments in which it is used. There are signs of a variable composition of the secretome depending on conditioning factors both external and inherent to the source cells, but the magnitude of this variability has not been accurately determined in the research carried out so far. Similarly, there is no unified consensus on the best methods to optimize and customize the secretome content, which is mandatory to achieve adaptable therapies with a broad spectrum of applications.

Another milestone that has not yet been reached is the development of finely tuned methods for secretome obtention. A great variety of methods for the isolation and purification of the whole secretome or EVs have been proposed, however, advances still need to take place to optimize the yield and scalability.

Moreover, as mentioned above, one of the pillars still under research is the development of secretome delivery strategies. The controlled and sustained release of secretome factors being the main goal, hydrogel encapsulation could be one of the most appropriate delivery strategies because of the simplicity and efficiency of the approach. Although EV encapsulation in hydrogels has lately been explored for tissue regeneration purposes, no studies have been published regarding MSC-secretome delivery devices for IMID treatment. Therefore, the study of this strategy is warranted in the near future. All these facts open a new promising avenue for the development of secretome-based therapies for the treatment of IMIDs.

## Figures and Tables

**Table 1:** Preclinical studies involving secretome application on inflammatory bowel disease (IBD), osteoarthritis (OA) and graft-versus-host disease (GvHD) animal models.

Disease	Secretome Fraction	Secretome Source	Preconditioning	Administration Route and Dose	Animal Model	Results	Ref.
Acute ulcerative colitis	Concentrated CM	AF-MSCs	-	Intraperitoneal injection of a 200 µL single dose	NOD-SCID mice	Decreased colon inflammation scores. TGFb1 and IL-10 increased levels. TNFα, IL1-b and MMP2 decreased levels.	[152]
Acute Ulcerative colitis	EVs	cA-MSCs	TNF-α and IFN-γ	Intraperitoneal injection of 100 µg at days 1, 3 and 5.	DSS-induced colitis mice	Decreased colon inflammation. Macrophage polarization trough M2 phenotypes. Treg enhanced expression in injured tissue.	[148]
Acute Ulcerative colitis	Exosomes	BM-MSCs	-	Intravenous injection of a non-determined dose	DSS/TNBS-induced colitis mice	Increased survival. Decreased inflammation scores. Increased levels of IL-10 in colon tissue due to higher production of M2 macrophages.	[149]
Acute Ulcerative colitis	EVs	hP-MSCs	-	200 µg single dose laparotomy injection	TNBS-induced colitis mice	Decreased oxidative stress. Improved colitis symptoms and its histopathological manifestations. Vesicle labelling showed a permanence of 2 days around administration area.	[150]
Acute Ulcerative colitis	EVs	Mouse BMSCs	-	Intraperitoneal injection of 50 µg per day for seven days	DSS-induced colitis mice	Macrophage M2 polarization was observed on EV-treated individuals as well as lower expression of proinflammatory cytokines such as IFN-γ, IL-12 or TNf-α	[151]
Acute GvHD	EVs	Human BM-MSCs	-	Tail vein injection of 1.6 × 10^6^ particles	Spleen cell injected B6D2F1mice	Suppression of the functional differentiation of T cells into CD8+ cells was observed in treated animals compared to control. EV injected animals showed mitigated clinical manifestation of disease and lower pathological damage in GVHD-targeted organs.	[167]
Chronic GvHD	Exosomes	hBM-MSCs	-	Tail vein injection of 100 µg once a week for 6 weeks	Bone marrow and spleen cell injected BALB mice	Activation of CD4+ T cells was suppressed avoiding their infiltration into disease targeted organs. Lower proinflammatory cytokines Th17, IL-22 and IL-21 production.	[168]
Acute GvHD	EVs	BM-MSCs	Co-culture with human platelet lysate	Intravenous injection of a non-specified EV dose	Human PBMCs injected xenograft mouse model	Overall survival of treated animals increased during the study. TNF-α and IFN-γ levels of treated individuals turned out to be lower compared to those of the control ones.	[139]
Acute GvHD	EVs	hUC-MSCS	-	Intravenous injection of 200 µg before and after disease induction	Allo-HSCT transplanted mice	Immunomodulatory effects of EVs were reflected through higher concentration of IL-10 and lower serum concentration of TNF-α, IFN-γ and IL-2	[166]
Acute GvHD	Exosomes	E-MSCs	-	Tail vein injection of 1 µg or 10 µg for 3 days	PBMC injected mice	Exosome treated mice show higher Treg levels compared to those treated with etanercept	[169]
Osteoarthritis	Conditioned media	UC-MSCs	3D culture	Intra-articular injection of 200 µL of concentrated CM per day for 4 days	Mycobacterium butyricum induced arthritis rats	Clinical manifestations of disease were milder in 3D-CM treated animals compared to 2D-CM treated ones. In vitro results of the study show the enrichment of the secretome in anti-inflammatory cytokines such as IL-10.	[125]
Osteoarthritis	Exosomes and MVs	Murine BM-MSCs	TGF-β	Intra-articular injection of single dose of 250 ng of exosomes or 500 ng of MVs	Collagenase induced mice	Prevention of cartilage degradation was observed in treated mice comparing to non-treated ones. Histological condition and damage was similar to healthy controls. Effects attributed to apoptosis prevention and macrophage differentiation inhibition exosomes being more efficient.	[154]

Abbreviations: AF-MSCs (amniotic fluid mesenchymal stem/stromal cells); cA-MSC (canine-adipose mesenchymal stem/stromal cells); DSS (dextran sodium sulfate); TNBS (2,4,6-trinitrobenzenesulfonic acid); BM-MSCs (bone marrow mesenchymal stem/stromal cells); hP-MSCs (human-placental mesenchymal stem/stromal cells); hUC-MSCs (human umbilical cord mesenchymal stem/stromal cells); PBMCs (peripheral blood mononuclear cells); HSCT (hematopoietic stem cell transplant); E-MSCs (embryonic mesenchymal stem/stromal cells); SM-MSCs (synovial membrane-derived mesenchymal stem/stromal cells).

**Table 2:** Studies involving hydrogel encapsulation of exosomes for increased treatment permanence around injured areas. MSC-EVs (mesenchymal stromal cell-derived extracellular vesicles) u-MSCs (umbilical-derived MSCs); p-MSCs (placenta-derived MSCs); a-MSC (adipose tissue-derived MSCs) e-MSC (embryonic stromal cells) 3D-a-MSC-secretome (3D cultured adipose-derived MSC-derived secretome).

Hydrogel Composition	Encapsulated Part of the Secretome	Model	Exosome Permanence and Effect	Ref.
Hyaluronic acid alginate	u-MSC-EVs	Critical-sized calvarial defect mice	Exosome released 14 days after treatment administration and improved bone regeneration	[208]
Silk fibroin	u-MSC-EVs	Hind limb ischemia mice	Sustained release after 36 days of administration and improved blood perfusion in injured areas	[214]
Peptides	u-MSC-EVs	Myocardial infarct rats	Sustained and steady release of exosomes until 26 days after administration and improved myocardial function	[215]
Methyl-cellulose/chitosan	p-MSC-EVs	Diabetic rats with cutaneous wound	Adhesive behaviour of the treatment around injured area and faster wound closure rates were observed	[195]
Alginate	a-MSC-EVs	Full thickness excisional wound rats	Hydrogel gradual degradation was observed and loaded exosome complete release was stated 172 h after treatment administration	[216]
Gelatin and laponite	3D-a-MSC-Secretome	Acute myocardial infarction rat models	Hydrogel shear-thinning behaviour was sought. Formulation injection was possible as well as its establishment into the peri-infarcted area.	[197]
Multidomain peptide nanofibers	e-MSC-secretome	Diabetes-induced kidney injury mice models	Hydrogel permanence on injected abdominal cavity was demonstrated was of 24 h. Secretome release was proven to be sustained and no burst release was observed when administered.	[198]
Silk fibroin	Bm-MSC-secretome	Age-related osteoporosis rat models	Release of bioactive factor was proven to be slow and continuous. Hydrogel matrix degradation and silk fibroin interaction with release medium was proposed to be responsible for this sustained release mechanism.	[203]
